# A Detailed Overview About the Single-Cell Analyses of Solid Tumors Focusing on Colorectal Cancer

**DOI:** 10.3389/pore.2022.1610342

**Published:** 2022-07-14

**Authors:** William J. Kothalawala, Barbara K. Barták, Zsófia B. Nagy, Sára Zsigrai, Krisztina A. Szigeti, Gábor Valcz, István Takács, Alexandra Kalmár, Béla Molnár

**Affiliations:** ^1^ Department of Internal Medicine and Oncology, Semmelweis University, Budapest, Hungary; ^2^ Molecular Medicine Research Group, Eötvös Loránd Research Network, Budapest, Hungary

**Keywords:** bioinformatics, colorectal cancer, multi-omics, heterogeneity, single-cell sequencing

## Abstract

In recent years, the evolution of the molecular biological technical background led to the widespread application of single-cell sequencing, a versatile tool particularly useful in the investigation of tumor heterogeneity. Even 10 years ago the comprehensive characterization of colorectal cancers by The Cancer Genome Atlas was based on measurements of bulk samples. Nowadays, with single-cell approaches, tumor heterogeneity, the tumor microenvironment, and the interplay between tumor cells and their surroundings can be described in unprecedented detail. In this review article we aimed to emphasize the importance of single-cell analyses by presenting tumor heterogeneity and the limitations of conventional investigational approaches, followed by an overview of the whole single-cell analytic workflow from sample isolation to amplification, sequencing and bioinformatic analysis and a review of recent literature regarding the single-cell analysis of colorectal cancers.

## Introduction

In 2012, researchers at The Cancer Genome Atlas Network published their work on the comprehensive molecular biological characterization of human colorectal cancers (CRC) [1]. They analyzed the exomes, copy number alterations, promoter methylation levels, transcriptomes, and microRNA fraction of bulk samples acquired from 276 patients with colorectal cancer. Since then, with the evolution of the equipment and toolbox of molecular biology with methods such as single-cell next generation sequencing (NGS), the need for an even more detailed investigation of organisms at a single-cell level has emerged. In this review article, we aimed to present contemporary methods and techniques for the sampling, isolation, and analysis of single cells and to give an overview of the current scientific literature about CRC at the single-cell level.

## Models for Tumor Heterogeneity

Tumor heterogeneity means that neoplastic cells from the same tumor can genotypically, phenotypically, morphologically, or metabolically differ from each other. The concept of heterogeneity has been around for several decades and gained attention in the 1990s when cancer stem cells were identified in acute myeloid leukemia [2]. There are two not mutually exclusive models explaining tumor heterogeneity: the cancer stem cell and the clonal evolution model. In the former model, tumor cells are hierarchically organized: a portion of cells, called the “stem cells” retain their ability to proliferate, while their offspring “differentiate” into nonproliferating cells [3]. The latter model describes cancer as a sequential process driven by somatic mutations following Darwinian mechanisms for subclonal selection [4]. Over the past decades, intratumoral heterogeneity has been intensively researched. Some cancer types (e.g., leukemias [2], breast cancer [5], brain tumors [6] and CRCs [7]) are thought to behave according to the cancer stem cell model, with evidence of a portion of cells being capable of inducing cancer in immunodeficient mice. Compelling evidence was found to the monoclonal origin and subclonal selection of several tumors including breast cancer [8], glioblastoma multiforme [9], and renal cell carcinoma [10].

## Tumor Microenvironment and Components of Tumors

Heterogeneity in solid tumors is not limited to the differences between neoplastic cancer cells. Cancerous cells are embedded into diverse tissues consisting of cancer-associated fibroblasts, extracellular matrix, vascular and lymphatic networks, and immune cells, among others. Cancer-associated fibroblasts, which have a constantly activated phenotype are the main components of tumor stroma [11]. Their exact origin and functions are not fully understood, but it is hypothesized that they can enhance tumor growth and progression, invasion, and metastatic potential as well [12]. They are more heterogeneous than normal fibroblasts and express various surface receptors and cytokines that facilitate tumor progression, angiogenesis, etc. [13]. Many tumors have been described to have marked immune cell infiltration. Some of these cells have antitumoral behavior (NK cells, CD8^+^ T cells, CD4^+^ Th1 cells, and APCs), while others can promote tumor progression (CD4^+^ Th2 cells, regulatory T cells, and tumor-associated macrophages) [13]. A meta-analysis published in 2020 found that high tumor-infiltrating lymphocyte (TIL) count with CD3^+^, CD8^+^ and FOXP3+ T-cells pose a prognostic benefit in CRC [14].

Another phenomenon that further expands heterogeneity of the tumor stroma is tumor budding and was described in several cancers including esophageal, pancreatic, endometrial, and breast cancer and was most extensively researched in CRCs. Tumor buds are isolated or small clusters of undifferentiated cancerous cells at the invasive front of the tumor tissue. The malignant cells in a tumor bud are morphologically different (loss of basal membrane, diverse shapes) from cells of the main tumor mass and express decreased epithelial and increased mesenchymal marker levels [15].

The extent of tumor heterogeneity has clinical implications as well. A recent study showed that sequencing of multiple tissue biopsy samples was able to detect more than twice as many mutations in solitary colorectal cancers compared to single tissue biopsy [16]. The genetic and epigenetic landscape of tumors influence tumor initiation, progression and drug response [17] thus the assessment of the degree of tumor heterogeneity may prove diagnostic and prognostic value and help treatment selection, monitoring of drug response and patient follow-up.

## Methods for Evaluating Tumor Heterogeneity

The above-mentioned characteristics of tumor heterogeneity explain the need for more sophisticated and sensitive methods for cancer cell biology research. Frequently used approaches for evaluating heterogeneity include various types of methods, such as immunohistochemistry, fluorescence *in situ* hybridization (FISH), comparative genome hybridization (CGH), microdissection combined with PCR, microarray techniques, etc. In 2005, Losi et al. confirmed the presence of intratumoral heterogeneity during the progression of CRC using microdissection and the above techniques, focusing on *p53* and *K-ras* mutations, and loss-of-heterozygosity on chromosomes 5q and 18q [18]. Their study concluded that prognostic and diagnostic genetic markers should be evaluated for heterogeneity as well.

The development and widespread use of NGS opened new paths towards understanding tumor heterogeneity more precisely. NGS is used for analyzing the genome, transcriptome, or accessible chromatin with techniques including DNA-seq, RNA-seq, or chromatin profiling methods, such as ChIP-seq. The sequence of a targeted gene panel, the exome (whole-exome sequencing, WES), or the whole genome (whole-genome sequencing, WGS) of multiple samples can be rapidly and relatively cost-effectively analyzed for single-nucleotide variations (SNVs) and copy-number variations (CNVs/SCNVs) with DNA-seq by fragmenting the genome into smaller pieces and sequencing them in parallel. Market leader companies in the genetic research industry, e.g., Illumina (San Diego, United States) offer several commercially available targeted gene panels for oncology including the field of both hematologic malignancies and solid tumors. Tumor samples can be sequenced in bulk or at a single cellular level with the evolving technical background. Bulk samples may contain several types of tissue including cancerous cells and their surrounding stroma, healthy surrounding tissue, smooth muscles, fat, and connective tissue. The sensitivity of bulk analysis is dependent on the coverage (average number of reads aligning to a known reference base) of the sequencing run and is typically between 5 and 10% [19]. This means that the detectability of a sought variant is highly dependent on its allele frequency, which in the field of oncology can be lower than the typical sensitivity of bulk sequencing. Thus, single-cell sequencing methods could be far more accurate and focused on characterizing intratumoral heterogeneity, however, the current isolation techniques are much more challenging and require designated equipment with relatively higher cost of reagents and subsequent analyses.

## Techniques for the Isolation of Single Cells

Numerous approaches have been developed for the isolation of single cells which differ in throughput, speed, cost, and efficiency. The starting sample material can be cell cultures, cell suspensions, or histopathologic slides.

The simplest method is termed limiting dilution. This technique is based on the dilution of cell suspensions and then aliquoting them into such volumes that it is statistically probable that a well contains only one cell [20].

Micromanipulation systems typically work with an inverted microscope and a motorized stage combined with glass micropipettes. Live, individual cells can be observed under the microscope and transferred to different compartments using the micropipettes [21]. The process is labour intensive manually but can be automatized with the help of computer vision and motorized stages [22].

Several microfluidics techniques exist for the separation of single cells. Cell suspensions can be separated through microchannels based on physical properties, immunomagnetic labelling or cell surface protein binding antibodies on the microfluidics chip [21]. 10x Genomics’ Chromium Controller (10x Genomics, Pleasanton, California, United States) solution offers a droplet-in-oil-based technique in which individual cells are encapsulated with uniquely barcoded beads thus enabling parallel sorting of cells and library preparation for NGS. The Bio-Rad ddSEQ Single-Cell Isolator (Bio-Rad Laboratories, Hercules, California, United States) offers a similar technique. Both platforms are capable of sorting and barcoding thousands of cells a day.

Fluorescence-activated cell sorting (FACS) is another high throughput method for separating individual cells. Cells bound with fluorescence-conjugated antibodies are passed through a flow cytometer and the antibodies are activated with laser beams. Detectors pick up scatter- and fluorescence signals from each cell which can then be individually diverted towards collecting compartments by an electromagnetic field based on their phenotype [23]. Penter et al. individually sorted cells applying this technique, and according to their results, the error rate was less than 1 out of 100 cells [24].

Magnetic-activated cell sorting (MACS) is an affinity-based cell sorting method. Antibodies conjugated with magnetic beads are bound to cells’ surface antigens. Cells are then placed in an external magnetic field, and after washing away unlabeled cells, the labelled cells can also be eluted [21].

Optical tweezers offer a procedure for non-contact cell separation using highly focused laser beams. Single cells can be selected, trapped and moved from one compartment to another with the help of optical forces [25].

**FIGURE 1:**
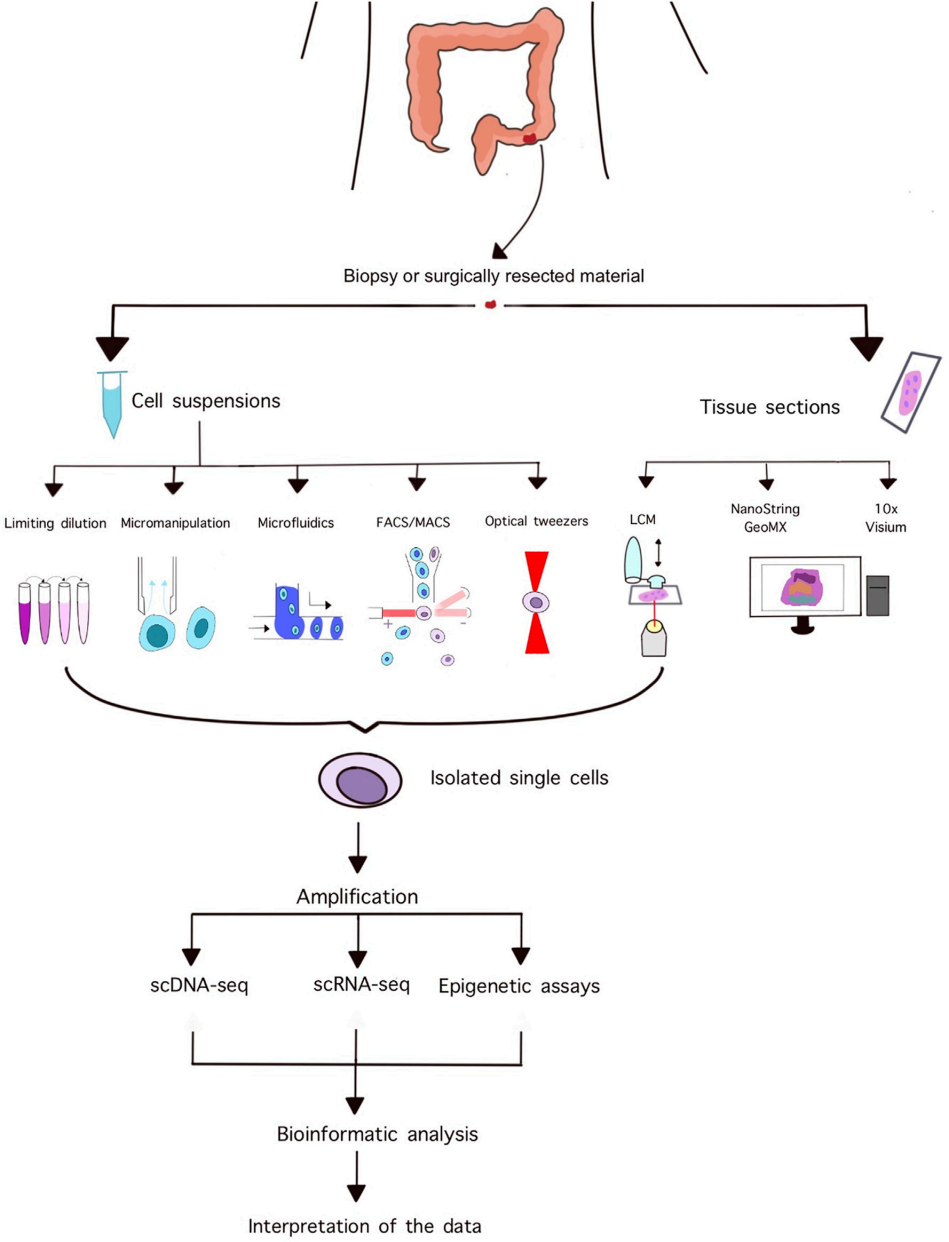
Summary of the workflow of single cell isolation and downstream analyses.

Laser capture microdissection is a popular technique for isolating homogeneous, uniform cell populations or even single cells from histopathological slides while simultaneously assessing tissue and cellular morphology. A typical instrument consists of an inverted microscope, a motorized stage, a laser unit, and a CCD camera [26]. The operator can manually adjust the power, speed, and focus of the laser, and can select preformed shapes or draw unique areas for dissection. Various methods exist for the subsequent isolation of dissected areas including gravitational forces pulling down the specimen to a collecting compartment, the use of adhesive-coated caps, or using a defocused laser beam to catapult the sample into the desired compartment [26]. These systems need to be manually supervised, moreover, working with single cells requires high operator skills due to the limited size of samples and lack of feedback systems. Figure 1 summarizes the workflow of single cell isolation, sequencing, and analysis.

## Molecular Biological Analysis of Single Cells

A typical eukaryotic cell contains ∼4 pg of genomic material while Illumina’s sequencing solutions need at least 1 ng of DNA for sequencing according to the manufacturer. Therefore, in eukaryotic single-cell sequencing at least a ∼1000-fold amplification is needed for subsequent analysis. This can be achieved by several methods including degenerate oligonucleotide-primed polymerase chain reaction (DOP-PCR), multiple displacement amplification (MDA), and multiple annealing and looping-based amplification cycles (MALBAC) among others. Some of these methods are PCR-based (e.g., MALBAC, DOP-PCR), while others use isothermal amplification (e.g., MDA).

Multiple displacement amplification utilizes the φ29 DNA polymerase, a high-fidelity enzyme with proofreading and strand displacement activity that works in an isothermal environment [27]. MDA uses random hexamer primers which offer great genome coverage and due to the enzyme’s strand displacement activity, multibranched DNA structures are generated. Its amplification is exponential, meaning small differences are disproportionally amplified causing sequence-dependent bias, producing over- and underamplified regions. As a result, this method is less effective in copy number variation (CNV) analysis than linear amplification methods, however, owing to the φ29 polymerase’s proofreading activity it is ideal for single nucleotide variation (SNV) detection [27].

Multiple annealing and looping-based amplification cycles is a PCR-based quasi-linear amplification technique. It utilizes the isothermal Bst DNA polymerase with strand displacement activity but does not have proofreading activity. The main advantage of MALBAC is that it only amplifies the original DNA template by using special primers that can form loops in full amplicons preventing them from serving as templates for another amplification cycle. After a few cycles of linear amplification, the product is further amplified with traditional PCR steps. The quasi-linear sense of this method makes it a great choice for CNV detection; however, it is less reliable for SNV detection due to the lack of the enzyme’s proofreading activity [28].

Degenerate oligonucleotide-primed *PCR* is another PCR-based amplification procedure using primers with a random hexamer sequence at the 3′ end and a fixed sequence at the 5′ end. In the first step, the random hexamer binds the genome and primer extension begins. Next, another set of primers specific to the 5’ end of the primers amplifies the products from the previous step. Thereby, DOP-PCR yields exponential amplification, yet, it is suitable for analyzing large CNVs [27].

Sequencing of RNA transcripts from single cells is also a possible approach. This requires the reverse transcription of RNA molecules to complementary DNA (cDNA) which can then be amplified and sequenced. To selectively target mRNA and exclude tRNA and rRNA, primers containing poly (dT) sequence binding the poly(A) tail of mRNA molecules are usually used for the reverse transcriptase enzyme generating cDNA [29]. After reverse transcription, cDNA can be amplified using several methods including PCR-based amplification [30] and *in vitro* transcription (IVT) using T7 RNA polymerase [31]. Amplified cDNA can then be subjected to library preparation and sequencing. Several protocols have been devised for single-cell RNA amplification and library preparation based on PCR amplification (Smart-Seq2 [32], SCRB-seq [33], DropSeq [34]) and IVT (MARS-Seq [35], inDrop [36], CEL-Seq [37]). Table 1 shows the advantages and limitations of different DNA and RNA amplification methods.

**TABLE 1:** Summary of DNA/RNA amplification methods.

Method	Enzyme used	Advantages	Limitations	References
DOP-PCR	*Taq* DNA pol	suitable for CNV detection with large bin sizes	Often yields low coverage, expontential amplification	[71]
MALBAC	*Bst* DNA pol	suitable for CNV detection	No proofreading activity, less reliable in SNV detection	[28]
MDA	*φ29* pol	proofreading activity	expontential amplification, less reliable in CNV detection	[72]
Homopolymertailing, PCR	*M-MuLV RT, TdT*, *Taq pol*	Captures truncated cDNAs as well [73]	Reduced coverage towards 3′ ends of transcripts, loss of strand information, exponential amplification	[30]
Template switching, PCR	*M-MuLV RT*, *Taq* pol	Maintains strand information, homogeneous transcript coverage	Lower sensitivity compared to homopolymer tailing, exponential amplification	[74]
*In vitro* transcription	*T7* RNA pol	Linear amplification	Each round shortens products [75], labor intensive	[37]

Epigenetic assays providing information about accessible chromatin and histone modifications such as bisulfite sequencing (BS-seq), chromatin immunoprecipitation sequencing (ChIP-seq), and assay for transposase accessible chromatin sequencing (ATAC-seq) are also available at a single-cell resolution. These methods offer a way to assess parts of the genome on a functional level by measuring DNA methylation level, open chromatin sites or histone modifications.

Bisulfite sequencing is an important method for DNA methylation analysis. Treatment of DNA with bisulfite salts converts unmethylated cytosines to uracil while methylated cytosines are spared allowing the assessment of DNA methylation at a single nucleotide level after sequencing. Clark et al. presented a protocol for single-cell BS-seq with which the methylation status of ∼50% of all CpG sites can be measured in single cells using post-bisulfite adaptor tagging, limiting the loss of adaptor-tagged sequences otherwise occurring during bisulfite treatment [38].

During ChIP-seq, protein-DNA complexes are cross-linked, followed by exonuclease-mediated DNA fragmentation. Fragmented DNA is then immunoprecipitated with antibodies specific to histone modifications or transcription factors allowing the sequencing of these target regions. Rotem et al. devised a way to perform single-cell ChIP-seq on multiple pooled cells to overcome the difficulties of the low input material from single cells [39]. They used a microfluidic system where fragmented chromatin from individual cells was uniquely barcoded by adapters and then pooled together for the immunoprecipitation step. After sequencing, the signal can be demultiplexed and fragments can be assigned to individual cells during the computational analysis.

Single-cell ATAC-seq was developed by Buenrostro et al. in 2015 using the Fluidigm C1 programmable microfluidic platform [40]. ATAC-seq uses a mutant hyperactive Tn5 transposase that identifies fragments and appends adaptors to nucleosome-free active regions. These tagged sequences are then purified and sequenced allowing the identification of active genomic regions [41].

Recently, two spatial transcriptomics platforms became commercially available: the 10X Visium Spatial Gene Expression and the NanoString Technologies’ GeoMX Digital Spatial Profiler (Seattle, United States). Although these platforms cannot yet reach exact single-cell levels, they add valuable histological and spatial information to the high-resolution transcriptomic data. 10X’s solution uses a special, oligo probe coated slide, where FFPE or fresh frozen tissue sections can be mounted. Staining and imaging are followed by tissue permeabilization, and the RNA molecules that have been released from cells bind to adjacent probes. The cDNA library constructed can then be sequenced and the probes are used to reconstruct spatial information of the sequenced transcriptome. NanoString’s solution works with tissue mounted on any type of glass slide. Targeted mRNA probes with unique barcodes joined by a photocleavable linker are hybridized to mRNA released from the tissue section which is also stained with fluorescent antibodies. After fluorescent imaging ROIs are selected where UV light cleaves the unique barcodes. Finally, the barcodes are sequenced and linked to unique mRNA targets mapping them to specific locations of the slide [42].

However, it is important to emphasize that the link between mRNA expression and protein translation is not always guaranteed [43], and genomic and transcriptomic studies should be validated at a protein level. Single-cell resolution analysis of proteins, such as single-cell flow cytometry and single-cell mass cytometry is also possible, the description of which is beyond the scope of this article.

## Bioinformatic Analysis of Single-Cell Sequencing Data

Sequencing instruments can produce several gigabytes of raw sequencing data which need to be processed and analyzed by a bioinformatics expert with sophisticated software tools and bioinformatic pipelines. A typical DNA-seq pipeline consists of quality control of the sequencing data followed by the alignment of reads to a reference genome. After this, variant calling can be performed to identify SNVs, and their allele frequencies compared to the reference genome. In single-cell analysis SNV allele frequencies should be close to 0.5 or 1 theoretically suggesting whether the cell is either heterozygous or homozygous to the SNV. However, because of the widely used non-linear amplification methods and the proportionally higher impact of artifacts occurring either before or during the early stages of the amplification step, the detected allele frequencies can deviate from the theoretically expected values [44]. To overcome this challenge, dedicated software tools such as SCAN-SNV measure amplification balance throughout the genome and calculate whether the detected allele frequencies are erroneous or not [44]. In bulk sequencing data, CNVs are called by measuring target read counts. Therefore, in single-cell analysis, the uniformity of genomic coverage needs to be taken into account during CNV calling [45]. Software tools for the analysis of CNVs in single-cell data include HMMCopy [46], AneuFinder [47], Ginkgo [48], and SCNV [49]. Mallory et al. conducted a performance assessment of popular single-cell CNV detection tools [45]. Table 2 lists examples of the software tools used in single-cell DNA-seq data analysis.

**TABLE 2:** Software tools for the bioinformatic analyses of DNA-seq data.

Tool	Usage	Reference
SCAN-SNV	Measures amplification balance, SNV detection	[44]
HMMCopy	CNV detection	[46]
AneuFinder	CNV detection	[47]
Ginkgo	CNV detection	[48]
SCNV	CNV detection	[49]

An RNA-seq pipeline starts with quality control of the raw data and is followed by read alignment, transcriptome reconstruction, expression quantification, and downstream analyses. Particularities of single-cell isolation techniques, such as doublet formation (two cells in the same oil droplet) or the capture of dead cells with droplet-based approaches must be considered during quality control of the raw data for which the ratio of transcripts/unique molecular identifier is widely used [50]. Read aligners can be splice-aware (TopHat [51], STAR [52]) or non-splice aware (BWA [53], Bowtie2 [54]), the former enabling larger gaps like those occurring at exon boundaries, while the latter does not allow such gaps. Transcriptome reconstruction aims to uncover all transcripts and their splice variants expressed in a sample [55]. This can be performed in either a reference-based manner where overlapping reference-aligned reads are used (Cufflinks) [56] or by *de novo* assembly, where an algorithm builds transcripts from short reads (SPAdes) [57]. Normalization techniques (e.g., median and quantile normalization) and gene-length corrections are usually used to reduce technical variation between samples and facilitate their comparison. The most widespread gene-length corrections are TPM (transcripts per million) and RPKM/FPKM (reads/fragments per kilobase per million reads). Several software packages are available for the normalization and differential expression analysis of single-cell RNA-seq data, including scran [58], SCnorm [59], TASC [60], and SCDE [61] (the detailed description of which is beyond the scope of this article). A performance comparison of normalization and differential expression analysis methods are summarized in Cole et al.’s [62] and Wang et al.’s work [63], respectively. Table 3 shows software tools for RNA-seq data analysis.

**TABLE 3:** Software tools for the bioinformatic analyses of RNA-seq data.

Tool	Usage	References
TopHat	Splice aware read aligner	[51]
STAR	Splice aware read aligner	[52]
BWA	Non-splice aware read aligner	[53]
Bowtie2	Non-splice aware read aligner	[54]
Cufflinks	Reference-based transcriptome reconstruction	[56]
SPAdes	*de novo* assembly	[57]
Scran	QC, normalization, complex analytic methods	[58]
SCnorm	Normalization	[59]
TASC	Differential expression analysis	[60]
SCDE	Differential expression, gene set overdispersion analysis	[61]

## Bulk vs. Single-Cell Experiments in Colorectal Adenomas and Adenocarcinomas

The comprehensive molecular characterization of CRCs by The Cancer Genome Atlas project identified several recurrent mutations, somatic copy number variations (SCNAs), DNA methylation patterns, and gene expression profiles and the integration of these findings has uncovered some key altered pathways deregulated during CRC formation and progression. The recurrently mutated genes included *APC*, *TP53*, *KRAS*, *PIK3CA*, *FBXW7*, *SMAD4*, *TCF7L2*, *NRAS*, *CTNNB1*, *SMAD2*, *SOX9*, *ATM*, *ARID1A*, and *FAM123B* in the non-hypermutated, and *ACVR2A*, *APC*, *TGFBR2*, *MSH3*, *MSH6*, *SLC9A9*, *TCF7L2*, and *BRAF* in the hypermutated tumors. Among SCNVs, chromosomal changes found included the gains of 1q, 7p and q, 8p and q, 12q, 13q, 19q and 20p and q with losses of 1p, 4q, 5q, 8p, 14q, 15q, 17p, and q, 18p and q, 20p, and 22q. Recurrent subchromosomal deletion peaks including *FHIT*, *RBFOX1*, *WWOX*, *SMAD4*, *APC*, *PTEN*, *SMAD3*, and *TCF7L2* were also observed. Furthermore, subchromosomal focal amplifications were detected in the case of *USP12*, *KFL5*, *CDK8*, *WHSC1L1*, *MYC*, *ERBB2*, *IGF2*, *INS*, and *TH* [1].

Since their integrative analysis, investigations using single-cell techniques have also been conducted to further evaluate intratumoral heterogeneity and clonal expansion in CRC. Table 4 presents a list of publications about single-cell analysis of colorectal cancers. Yu et al. performed scWES on cells isolated by micropipetting from a single-cell suspension of cancerous and normal adjacent tissues of colon cancer patient in 2014 [64]*.* Population genetics and potential driver events were investigated in 63 single tumor cells and compared to the results of the bulk sequencing data of 21 colon cancer patients. They identified two independent clones in the tumor cell population with the major clone containing *APC* and *TP53* mutations, which were absent in the minor clones harboring mutations in *CDC27* and *PABPC1* genes, indicating biclonality in CRC. They also identified a potential driver event, the frequent mutation of *SLC12A5* in single tumor cells, showing how single-cell sequencing can provide insight into rare genetic events otherwise masked by the whole population. In 2017 Wu et al. studied the heterogeneity and evolution of non-hereditary CRC in two patients by combining bulk WES with scWES [65]. Normal polyps, adenomatous polyps, CRC, and matched normal mucosa acquired via biopsy were in part sequenced in bulk, while the other part was digested into cell suspensions from which single cells were isolated by a micromanipulation system. By comparing the results of bulk WES with scWES, they found that bulk sequencing underestimated the level of heterogeneity of the tissues compared to single-cell analyses, and with scWES, they were also able to cluster the cells. Based on their results they proposed a monoclonal origin of CRC. In 2018 Roerink et al. used immortalized clonal organoids as proxies for the single cells obtained by flow-sorting normal and cancerous colorectal stem cells [66]. Their argument for choosing this method was that using true single cells with the contemporaneous amplification techniques would result in incomplete coverage and artefactual SNV calling. By profiling the SNVs, mutational patterns, methylome, transcriptome, and drug response of clonal organoids derived from 4–6 tumor sites and matching normal tissue from 3 colorectal patients they were able to describe intratumoral heterogeneity and possible phylogeny of these tumors. In the same year, Bian et al. investigated FACS/MACS sorted single cells obtained from multiregional samples of surgically resected material of 10 CRC patients using scTrio-seq2 method. This technique can simultaneously assess SCNVs, methylation level, and also the transcriptome of cells [67]. After investigating cells from multiple sites including the primary tumor, lymph node metastases, liver metastases, and posttreatment liver metastases and identifying sublineages based on subclonal SCNAs within chromosome arms assessed intratumoral heterogeneity and the dynamics of DNA methylation and gene expression. Zhou et al. investigated single cells of CRC patients and elderly cancer-free individuals using parallel single-cell genome and transcriptome sequencing. They included 21 CRC patients with primary tumor and matched normal mucosal samples with peripheral blood, adjacent lymph nodes and mesenteric blood vessel samples also obtained from 12, 4, and 4 of those patients, respectively. Six elderly cancer-free individuals’ peripheral blood samples were also obtained. After digesting the samples into cell suspensions and sorting them by FACS based on surface markers, they performed genome and transcriptome sequencing. Analyzing the SCNA profile of the cells they concluded that every cell type, even the immune cells isolated from cancer-free individuals’ blood contained SCNAs, mostly deletions of X chromosome in females and Y chromosome in males. Fibroblasts isolated from primary tumors had the highest percentage of SCNAs (as high as 48% of cells) with frequent gains of the whole chromosome 7. These cells, compared to fibroblasts isolated from normal adjacent tissues had 76 differentially expressed genes (DEG), 5 of which (*BGN*, *RCN3*, *TAGLN*, *MYL9*, and *TPM2*) were associated with poorer prognosis in the TCGA database [68]. Wang et al. performed droplet-based scRNA-seq on cancerous and adjacent non-malignant inflamed tissue from a patient with ulcerative colitis-associated colon cancer [69]. They analyzed 2250 cells from tumor tissues and 2527 cells from non-malignant tissues, and classified them into cell types (myeloid cells, T cells, B cells, fibroblasts, endothelial cells, and epithelial cells) based on their transcriptional activity and further clustered them using the t-SNE method. This enabled to compare gene expression activity between malignant and non-malignant derived cells among each cell types, characterizing the tumor microenvironment, and found that many malignant clusters presented protumoral activity. They also performed pseudotime analysis to evaluate the development of ulcerative colitis (UC) to colitis-associated colon cancer (CAC), and found that CD74, CLCA1 and DPEP1 may play a key role in disease progression. Recently, Liu et al. analyzed four cohorts containing gene expression data and developed a prognostic model based on immune cell type composition of colorectal cancers [70]. They were able to associate several immune cell subtypes with the prognosis of patients, such as a subgroup of dendritic cells with better, and certain subgroups of macrophages, and B cells with poorer prognosis. One of the cohorts analyzed contained scRNA-seq data, and they evaluated the ratio of these subtypes in the tumor microenvironment of these samples, showing how single-cell analyses may prove prognostic value in oncologic patient care.

**TABLE 4:** Overview of single-cell CRC publications and their findings.

Year	Method	Findings	References
2014	scWES	Observed biclonality in CRC, identified a rare driver mutation at single-cell level with low prevalence at the population level (SLC12A5)	Yu et al. [64]
2017	scWES	Proposed a monoclonal origin of CRCs	Wu et al. [65]
2018	Bulk sequencing of organoids derived from single cells	Assessment of intratumoral heterogeneity and phylogeny of cells	Roerink et al. [66]
2018	scTrio-seq2	Successful multi-omics characterization of ∼1900 single cells of 12 CRC patients	Bian et al. [67]
2020	Parallel single-cell genome and transcriptome sequencing	Identification of SCNAs present in more than 10000 cells, DEGs in fibroblasts from tumor compared to fibroblasts from NAT	Zhou et al. [68]
2021	scRNA-seq	Found protumoral gene expression activity in tumor-derived cells in different cell types, proved insights into progression of UC to CAC	Wang et al. [69]
2022	Analysis of scRNA-seq, RNA-seq and microarray cohorts	Built a prognostic model based on immune cell type composition, analyzed the immune cell subgroups in the TME	Liu et al. [70]

## Conclusion

Single-cell genomic, transcriptomic, and epigenetic methods are powerful tools in cancer cell biology research. With these methods, intratumoral heterogeneity and cancer evolution can be investigated in unprecedented detail, unveiling otherwise averaged out cell populations, identifying driver events, and understanding cancer phylogenetics. In the forthcoming era of precision medicine, single-cell analyses will be essential for a more detailed understanding of cancer formation, progression, and metastatic spread. Moreover, by identifying therapy-resistant clones and potential sensitivity to treatments the above-mentioned techniques will provide a tool for clinicians to administer the best possible treatment regimen to patients.
